# Health-promoting behaviors among patients with heart failure: A cross-sectional study in Georgia

**DOI:** 10.21542/gcsp.2025.33

**Published:** 2025-06-30

**Authors:** Tengiz Verulava, Revaz Jorbenadze

**Affiliations:** 1 Health Policy Institute, Caucasus University, Tbilisi, Georgia; 2 School of Health and Biomedical Sciences, RMIT University, Melbourne, Australia; 3 Chapidze Emergency Cardiology Center, Tbilisi, Georgia

## Abstract

**Background:** The increase in the number of patients with heart failure in recent years is multifactorial and is partly due to an aging population, improved survival from acute cardiovascular events (e.g., myocardial infarction), and advances in primary and secondary prevention strategies that have extended life expectancy of patients.

**Objective:** The purpose of this study is to evaluate health-promoting behaviors in patients with heart failure.

**Methodology:** Using a cross-sectional, observational research method, 143 patients diagnosed with heart failure at the Chapidze Emergency Cardiology Center (Tbilisi, Georgia) were interviewed. The average age of the patients was 64.8 ± 9.2 years, 63% of whom were men.

**Results and discussion:** The results showed a negative association between age and healthy eating habits, as well as a positive mental attitude. Higher education levels were associated with better health behaviors. The more knowledge patients had about their disease, the more likely they were to engage in health-promoting behaviors. Patients with a disease duration of >15 years were more likely to engage in healthy eating habits and maintain a positive mental attitude. Subjects implanted with a pacemaker/cardioverter had a low-confidence mental attitude.

**Conclusions:** Interventions that promote healthy behaviors in patients with heart failure should be expanded, particularly in those who are older, less educated, have a longer disease duration, have low disease knowledge, and/or are implanted with a cardioverter/pacemaker. Future interventions should include educational, psychological, and behavioral support strategies tailored to individual needs to encourage health-promoting behaviors and maintain a positive mental attitude among patients with HF.

## Introduction

Heart failure is a chronic condition in which the heart becomes weakened or stiff, making it unable to pump sufficient blood to meet the oxygen and nutrient demands of the body’s tissues and organs. Heart failure is a significant global health issue that affects over 64 million people worldwide. It represents a leading cause of morbidity, mortality, and healthcare expenditure^1^. The increase in the number of patients with heart failure in recent years is multifactorial and is partly due to an aging population, improved survival from acute cardiovascular events (e.g., myocardial infarction), and advances in primary and secondary prevention strategies that have extended life expectancy^2^. Consequently, heart failure has become a chronic condition that is managed over the long term rather than a terminal stage of acute cardiac disease.

The incidence of heart failure varies with age and sex^3^. Studies have shown that hospitalization rates for heart failure are high among individuals aged ≥ 65 years^4^. In elderly patients, low physical activity, depression, and anxiety increase the risk of heart failure and worsen its clinical outcomes^5^. The prevalence of heart failure is relatively higher in men compared to women^6^.

Heart failure is a chronic, life-threatening disease that tends to progressively worsen over time. Due to its unfavorable outcomes, half of all patients with heart failure die within four years, and in severe cases, 50% of patients die within one year^7^. It is the final stage of almost all cardiovascular diseases.

Despite the unfavorable prognosis, with a healthy lifestyle and proper treatment, patients with heart failure can maintain good clinical and functional status, as well as social activity^8–10^. Health behaviors are some of the most important determinants of health. Adopting healthy behaviors has a profound impact on health, well-being, and overall quality of life^11–13^. Health behaviors include actions, activities, and habits related to improving, maintaining, or restoring health. These behaviors can be both health-promoting and health-risking.

Health-promoting behaviors, such as maintaining a nutritious diet and engaging in regular physical activity, play a vital role in preventing chronic diseases, boosting energy levels, and enhancing overall well-being. On the other hand, risky behaviors like consuming an unhealthy diet, smoking, and excessive alcohol consumption can increase the likelihood of developing heart disease, diabetes and obesity^14^.

Healthy behaviors and medication play a significant role in improving the health of patients with heart failure^15^. Practicing healthy behaviors means changing unhealthy habits to adopt and maintain a healthier lifestyle.

The aim of our study is to evaluate the health behaviors of patients with heart failure, focusing on both health-promoting and risky behaviors. Understanding and promoting these behaviors through effective intervention strategies is essential for improving disease outcomes and patients’ quality of life.

### Methodology

**Study design and participants**

A cross-sectional observational study was conducted between January and August 2024 at the Chapidze Emergency Cardiology Center in Tbilisi. Ethical approval was obtained from the Caucasus University Bioethics Agency (CU: 18/I/2024).

Inclusion criteria:

• Aged between 55 and 80 years,
• Confirmed diagnosis of heart failure (based on ESC guidelines),
• Ability to understand and complete the questionnaire, and
• Provision of informed consent.

Exclusion criteria:

• Severe cognitive impairment,
• Acute decompensated heart failure at the time of the survey,
• Hearing or vision problems that interfered with participation,
• Refusal to participate.

Out of 165 eligible patients approached during the study period, 143 agreed to participate, yielding a response rate of 86.7%. No significant demographic differences were observed between responders and non-responders.

**Health behavior questionnaire**

Health behavior was assessed using the Health Behavior Inventory, consisting of 24 items grouped into four categories^16^:

1. Healthy Diet Habits –evaluates dietary behavior, emphasizing consumption of fruits and vegetables, balanced nutrition, and avoidance of foods high in sugar, animal fats, and salt.
2. Preventive Behaviors: Assesses adherence to health recommendations, awareness of health and illness, regular health screenings, compliance with medical advice, and proactive health information-seeking.
3. Positive Mental Attitude: Evaluates the level of positive perception and avoidance of excessive emotions, tension, stress, and depressive situations.
4. Healthy Practices: Includes physical activity and rest behaviors such as adequate rest, avoiding excessive work, body weight control, and abstaining from tobacco and excessive physical exertion.

Responses were recorded on a 5-point Likert scale (1 = “rarely” to 5 = “almost always”). Subscale scores were summed to yield a total health behavior index ranging from 24 to 120, with higher scores indicating more favorable health behaviors. The scale demonstrated good internal consistency (Cronbach’s *α* = 0.85).

### Sociodemographic questionnaire

The sociodemographic section included 18 items covering age, gender, education level, marital status, disease duration, family history of cardiovascular disease, adherence to medical recommendations, tobacco and alcohol use, and self-reported physical and mental well-being. Responses were recorded on a 5-point scale (1 = “very bad” to 5 = “excellent”).

### Statistical analysis

Descriptive statistics (means and standard deviations) were calculated using PSPP and Microsoft Excel 2020. To account for Type I error inflation from multiple comparisons (5 outcome variables × 8 predictors = 40 tests), a Bonferroni correction was applied. The adjusted significance threshold was set at *α* = 0.00125. Associations with corrected *p*-values below this threshold were considered statistically significant; others were interpreted cautiously as exploratory.

A post hoc power analysis was conducted using assumptions based on G*Power software. For independent samples t-tests with a medium effect size (Cohen’s *d* = 0.5), *α* = 0.05, and a total sample size of 143 (approximately 71 per group), the estimated statistical power was 84%. This indicates the study was adequately powered to detect medium-sized effects, though underpowered to detect small effects, particularly in subgroup analyses.

To address missing data, cases with substantial missing responses (more than 10% of items unanswered) were excluded from the analysis. For isolated missing items (affecting less than 5% of participants), mean substitution within the relevant subscale was applied to preserve the overall dataset. Sensitivity analyses showed that this approach did not significantly alter the main outcomes.

## Results

A total of 143 patients participated in the research, 63% of whom were men (*n* = 90) and 37% were women (*n* = 53). The age of the respondents ranged from 55 to 74 years, with an average age of *M* = 64.8 years (SD = 9.2). Most respondents had a secondary education (*n* = 101; 71%), were married (*n* = 107; 75%), and lived in an urban area (*n* = 84; 59%).

Among the participants, 31% (*n* = 45) had an illness length of more than fifteen years, and 63% had an implanted heart device. Family members of 43% (*n* = 61) of respondents also had cardiovascular diseases, and 29% (*n* = 42) had been hospitalized in the past year. Additionally, 35% of patients (*n* = 51) did not follow their doctor’s recommendations, and 24% (*n* = 34) were regular tobacco users (see Table 1).

**Table 1:** Sociodemographic and health characteristics of patients (*n* = 143).

	*n*	%
Age (years) (mean ± SD)	–	64.8 ± 9.2
**G** **ender**		
Woman Man	53 90	37% 63%
**P** **lace of residence**		
City Village	84 59	59% 41%
**Education level**		
Secondary Higher	101 42	71% 29%
**Marital status**		
Married Lonely	107 36	75% 25%
**D** **isease** ** duration**		
<1 year 1–5 years 6–10 years >10 years	25 31 42 45	18% 22% 29% 31%
**F** **amily** ** history of CVD**		
Yes No I don’t know	61 47 35	43% 33% 24%
**Hospitaliz** **ed** ** last year**		
Yes No	42 101	29% 71%
**Follow** **s** ** doctor’s recommendations**		
Yes No	92 51	65% 35%
**Tobacco** ** use**		
Yes No	34 109	24% 76%

The mean health behavior score of the patients was 80.20 (SD = 18.22). Among the health behavior categories, healthy practices had the highest score (*M* = 3.45, SD = 0.86), followed by positive mental attitude (*M* = 3.39, SD = 0.95), healthy eating habits (*M* = 3.36, SD = 1.18), and preventive behaviors (*M* = 3.35, SD = 0.89) (see Table 2).

**Table 2:** Health Behavior Index survey results (*n* = 143).

	Mean	Standard deviation	Minimum	Maximum	Median
Healthy eating habits	3.36	1.18	1.13	5.65	3.12
Preventive behavior	3.35	0.89	1.67	4.87	3.40
Positive mental attitude	3.39	0.95	1.35	4.94	3.51
Health practices	3.45	0.86	1.57	4.58	3.24
General health behavior	80.20	18.22	48.2	121.00	75.00

**Table 3:** Health behaviors and sociodemographic variables.

Variables	PEH	p	PB	p	PNA	p	HP	P	HBI	p
**G** **ender**										
Woman	24,5 ± 3,1	0,0001	25,2 ± 3,6	0,0004	21,9 ± 3,7	0,3044	24,8 ± 2,9	0,0028	92,3 ± 11,1	0,0005
Man	18,2 ± 5,1		18,8 ± 3,4		20,4 ± 3,6		19,7 ± 4,3		78,5 ± 15,1	
**Age**										
55–65	18,62 ± 4,5	0,0018	19,19 ± 3,8	0,0479	18,76 ± 3,31	0,1714	18,97 ± 4,52	0,1263	80,14 ± 13,5	0,0086
66–75	20,72 ± 5,2		20,57 ± 4,4		20,76 ± 3,73		20,16 ± 5,18		82,67 ± 13,67	
≥76	22,91 ± 3,85		22,13 ± 3,8		23,08 ± 3,11		23,66 ± 3,47		89,67 ± 9,73	
**Education**										
Average	18,3 ± 4,3	0,0894	18,6 ± 4,95	0,7985	19,65 ± 4,73	0,1732	19,85 ± 2,83	0,1732	78,31 ± 15,8	0,2937
Higher	25,87 ± 4,94		25,92 ± 3,76		24,93 ± 3,48		24,92 ± 4,83		88,32 ± 16,78	
**Marital status**										
Married	18,28 ± 5,16	0,3638	18,13 ± 4,3	0,6274	18,38 ± 3,24	0,0534	18,67 ± 4,24	0,4713	78,29 ± 16,5	0,3832
Single	18,28 ± 5,16		18,13 ± 4,3		18,38 ± 3,24		18,67 ± 4,24		78,29 ± 16,5	
**D** **uration of the disease**										
>1 year	17,12 ± 4,8	0,1248	17,91 ± 5,2	0,4831	17,82 ± 3,8	0,8319	17,11 ± 3,8	0,3727	78,32 ± 12,8	0,7562
1–5 years	20,2 ± 4,6		19,76 ± 4,7		19,13 ± 3,1		19,51 ± 4,2		81,93 ± 12,8	
5–10 years	21,15 ± 4,6		21,81 ± 4,52		21,82 ± 3,9		22,83 ± 3,9		84,84 ± 11,93	
<10 years	22,91 ± 5,3		23,21 ± 3,5		23,84 ± 4,1		23,78 ± 4,7		86,39 ± 14,43	
**Hospitalization last year**										
Yes	22,31 ± 4,7	0,1371	21,61 ± 4,6	0,6429	22,94 ± 4,7	0,8941	22,83 ± 4,9	0,352	84,94 ± 12,83	0,7891
No	19,45 ± 3.6		18,11 ± 3,3		18,56 ± 3.8		17,98 ± 4,6		80,27 ± 12,53	
**Tobacco consumption**										
Yes	18,56 ± 4,87	0,0001	18,77 ± 6,12	0,0005	19,56 ± 3,56	0,8832	18,63 ± 4,45	0,0001	70,6 ± 18,14	0,0001
No	21,57 ± 3,67		22,89 ± 3,23		22,67 ± 3,69		22,67 ± 2,12		88,34 ± 9,4	
**Following the doctor’s recommendations**										
Yes	20,35 ± 3.96	0,0001	23,45 ± 4,10	0,0005	23,46 ± 4,31	0,0004	26,25 ± 4,18	0,0001	90,86 ± 12,57	0,0001
No	16,45 ± 4,5		15,76 ± 5,62		18,83 ± 3,32		19,14 ± 4,89		73,51 ± 14,38	
**Health behavior index in patients with an implanted pacemaker/** **cardioverter**										
Yes	19,25 ± 3.87	0,0001	20,45 ± 3,89	0,0005	17,23 ± 4,12	0,0004	20,25 ± 4,28	0,0005	72,41 ± 12,38	0,0001
No	19,45 ± 4,1		20,85 ± 4,72		25,56 ± 5,11		19,90 ± 4,78		91,76 ± 11,13	

**Notes.**

M ± SD mean ± standard deviation PEH correct eating habits PB preventive behavior PNA positive mental attitude HP Health Practice HBI Healthy Behavior Inventory

A significant correlation was observed between patients’ health behaviors and their age. As age increased, so did the health behavior index (89.67 ± 9.73). This relationship was consistent across all health behavior categories.

Women had a higher health behavior score (86.39 ± 14.43) compared to men. Specifically, women demonstrated better eating habits (24.5 ± 3.1), preventive behaviors (25.2 ± 3.6), health practices (24.8 ± 2.9), and an overall health behavior inventory score (92.3 ± 11.1).

The study also showed that patients with higher education had better health behavior scores across multiple categories compared to those with lower education. This included correct eating habits (25.87 ± 4.94), preventive behaviors (25.92 ± 3.76), positive mental attitude (24.93 ± 3.48), health practices (24.92 ± 4.83), and overall health behavior (88.32 ± 16.78).

Patients who adhered to medical recommendations had a higher health behavior score (90.86 ± 12.57) compared to those who did not follow recommendations (73.51 ± 14.38).

Heart failure patients with a disease duration of more than 10 years had higher health behavior scores (86.39 ± 14.43) compared to patients with a shorter disease duration.

The influence of health behaviors and adherence to health recommendations plays a central role in reducing hospitalizations and mortality among patients with heart failure. The study highlighted that patients who followed medical recommendations had higher health behavior scores (90.86 ± 12.57) compared to those who did not (73.51 ± 14.38).

Finally, patients with heart failure who did not have a pacemaker implanted demonstrated more health behaviors in the positive mental attitude category (25.56 ± 5.11) and overall health behavior score (91.76 ± 11.13).

## Discussion

The survey showed the average level of health behavior among the patients. A positive mental attitude received the maximum ratings, followed by healthy eating habits, preventive behaviors, and health practices (Table 3).

Health behavior outcomes in our study were lower than those reported in other studies. Several factors may explain why health behavior outcomes in our study were lower than in other studies. First, socioeconomic factors such as lower education levels, limited access to health information, and financial constraints may limit patients’ ability to adopt and maintain healthy behaviors. Additionally, the majority of participants in our sample had only secondary education, which may be associated with lower health literacy and self-efficacy. Cultural factors and differences in health system infrastructure between countries may also influence patient engagement in health-promoting behaviors. Furthermore, our study relied on self-reported data, which may reflect more realistic, less socially desirable responses compared to other studies where patients might have over-reported positive behaviors.

While several associations appeared statistically significant before correction, Bonferroni adjustment reduced the number of significant findings. Only a few associations, primarily those with very small original *p*-values (e.g., *p* = 0.0001), remained statistically significant after correction. This highlights the importance of cautious interpretation, especially for marginal results, and underscores the exploratory nature of the study.

Furthermore, the absence of a formal sample size calculation represents a limitation of the study. This restricts our ability to determine whether non-significant results reflect a true lack of association or insufficient statistical power. Future studies should conduct power analyses based on expected effect sizes to ensure appropriate sample sizes for detecting meaningful differences.

Gender and age meaningfully affect the level of health behavior, while place of residence and marital status do not have a significant impact. Our findings confirm that, within our sample, women demonstrated higher levels of health behavior compared to men. In particular, correct eating habits, preventive behaviors, health practices, and the overall health behavior inventory were higher in women. The same results were detected in other studies^17^.

Our research confirmed that age correlates with health behaviors in the categories of healthy eating habits and positive mental attitude. As age increases, health behaviors tend to decrease.

Research has also shown that education levels influence health behaviors. As education levels increase, so do activities related to health behavior. The results are consistent with other studies that show education increases health behaviors^18,19^. Additionally, people with higher education are more motivated to obtain information related to their disease and are more likely to adapt to it. Our study indicates that education positively affects health behavior.

Our study also established a link between the duration of chronic illness and patients’ health behaviors. This relationship can be attributed to the increased knowledge patients acquire over time regarding proper nutrition and preventive strategies. The longer the disease duration, the higher the health practice index. This suggests that patients actively participate in the treatment process, seeking more knowledge about their condition. Other studies also confirm that patients with a longer illness follow health behaviors more closely than those with a shorter illness. However, some studies show the opposite, indicating that the longer the duration of the disease, the lower the health behavior^20,21^. This could be due to the increased severity of the disease, greater dependence on others, higher levels of anxiety, and feelings of loneliness, frustration, and low confidence that can reduce positive mental attitudes and hinder health behaviors.

Adherence to physicians’ prescriptions is critical for patients with heart failure. A concerning 35% of patients in our study did not follow their doctor’s recommendations, highlighting a significant gap in adherence that may negatively impact disease management and outcomes. This finding underscores the need for interventions aimed at improving patient compliance and education.

The study showed that patients who adhere to medical recommendations have a higher rate of health behavior (90.86 ± 12.57) and health practice (26.25 ± 4.18). Elderly individuals in our study tended to follow doctor’s orders more frequently. This may be influenced by a combination of factors such as increased health awareness, greater experience with the healthcare system, a stronger sense of responsibility for their health, and potentially more available time to attend appointments and follow medical routines^22^. However, this association is likely multifactorial and not solely attributable to retirement or increased free time.

The development of cardiovascular diseases is negatively affected by tobacco consumption^23–25^. By incorporating health behaviors into daily practice, increased awareness can help prevent further disease progression. According to our research, 24% of respondents are regular tobacco users. Our study confirmed that non-smokers achieve better health behavior outcomes (88.34) compared to smokers (70.6). Non-smoking respondents scored higher in all health behavior categories. The results align with those of other studies^26^.

According to researchers, the implantation of a pacemaker improves patients’ emotional well-being. However, other studies indicate that patients with implanted pacemakers are more likely to experience emotional difficulties that interfere with their daily lives. Our study confirmed that pacemaker/cardioverter implantation reduces positive mental attitude (17.23 ± 4.12). This suggests that these patients may experience anxiety because they feel they have exhausted their treatment options.

To improve health-promoting behaviors and mental attitudes in patients with heart failure, multifaceted interventions are necessary. Education-based programs tailored to patients’ literacy levels can enhance disease knowledge and motivate lifestyle changes. Personalized counseling by nurses or health coaches has shown effectiveness in fostering behavior change, particularly regarding diet, physical activity, and medication adherence. Additionally, integrating cognitive-behavioral strategies and stress management techniques, such as mindfulness, relaxation training, or peer support groups, can improve emotional resilience and help maintain a positive mental attitude. Technology-assisted tools, like mobile health apps and reminder systems, can further support behavior monitoring and adherence.

## Conclusions

The health behavior of patients with heart failure is influenced by factors such as gender, age, education, marital status, and disease duration. The study revealed relationships between individual categories of health behaviors and adherence to medical recommendations. Health behaviors in patients with heart failure are rated higher among women, non-smokers, individuals with higher education, and married persons.

It is recommended to expand interventions that promote health behaviors in patients with heart failure, particularly for those who are older, less educated, have a disease duration of more than 15 years, have low disease knowledge, and/or have a cardioverter/pacemaker implanted.

Future interventions should include educational, psychological, and behavioral support strategies tailored to individual needs to encourage health-promoting behaviors and maintain a positive mental attitude among patients with HF.

## Ethical approval

Before starting the study, we received approval from the Research and Ethics Committee of Caucasus University (CU 39-21.01.24.). The research participants were informed about the research issue, their participation was completely voluntary and they could refuse to participate in the research at any time.

## Competing interests

The authors declare no competing interests relevant to the content of this article.

## Funding:

The authors received no funding for the present study.

## Author contribution

All authors contributed equally to this work.
